# The Potential Anticancer Activity of Phytoconstituents against Gastric Cancer—A Review on In Vitro, In Vivo, and Clinical Studies

**DOI:** 10.3390/ijms21218307

**Published:** 2020-11-05

**Authors:** Sylwia Nakonieczna, Aneta Grabarska, Wirginia Kukula-Koch

**Affiliations:** 1 Chair and Department of Pharmacognosy, Medical University of Lublin, 1, Chodzki str., 20-093 Lublin, Poland; sylwia.nakonieczna2@gmail.com; 2 Chair and Department of Biochemistry and Molecular Biology, Medical University of Lublin, 1, Chodźki, 20-093 Lublin, Poland

**Keywords:** phytotherapy, gastric cancer, phytocompounds, natural medicine

## Abstract

Gastric cancer belongs to the heterogeneous malignancies and, according to the World Health Organization, it is the fifth most commonly diagnosed cancer in men. The aim of this review is to provide an overview on the role of natural products of plant origin in the therapy of gastric cancer and to present the potentially active metabolites which can be used in the natural therapeutical strategies as the support to the conventional treatment. Many of the naturally spread secondary metabolites have been proved to exhibit chemopreventive properties when tested on the cell lines or in vivo. This manuscript aims to discuss the pharmacological significance of both the total extracts and the single isolated metabolites in the stomach cancer prevention and to focus on their mechanisms of action. A wide variety of plant-derived anticancer metabolites from different groups presented in the manuscript that include polyphenols, terpenes, alkaloids, or sulphur-containing compounds, underlines the multidirectional nature of natural products.

## 1. Introduction

Gastric cancer (GC) is one of the most common cancer types and a leading cause of death worldwide. According to the World Health Organization (WHO) reports, GC is ranked fifth for cancer incidence and third for cancer deaths [1]. It occurs predominantly in countries such as Japan, Korea, and China [2] and is more common among males than females [3]. The National Cancer Institute emphasizes that although death rate for GC has declined, the number of this type of cancer cases has been continuously increasing in the last decade.

Gastric cancer, as many carcinomas, is highly heterogeneous at the cellular and molecular level. Majority of GCs called adenocarcinoma arise from glandular epithelia of the gastric mucosa [4]. The two major histological types of gastric adenocarcinoma, such as intestinal and diffuse, have been described by Lauren [5]. While the diffuse type of gastric cancer is more aggressive and its pathogenesis remains still unclear, the intestinal type of adenocarcinoma has a better prognosis. The latter is often found in older patients and its occurrence and development is more associated with environmental factors, lifestyle choices (smoking, regular alcohol consumption, and frequent intake of smoked and mostly pickled foods with a high content of salt), and chronic inflammation induced by *Helicobacter pylori* infection [6]. Moreover, understanding of many genetic and molecular changes identified in GC in recent years, including activation of oncogenes, overexpression of growth factors/receptors, inactivation of tumor suppression genes, DNA repair genes and cell adhesion molecules, loss of heterogeneity and point mutations of tumor suppressor genes, and silencing of tumor suppressors by CpG island methylation, has been helpful in both development of new tumor classification, its prevention and clinical practice to improve the overall survival [7].

Currently, the standard therapeutic procedures for GC patients include surgical resection of tumor, chemotherapy, radiotherapy, or chemoradiotherapy [8]. However, despite significant advances in medicine, GC is still being seen as public health issue, mainly due to its late diagnosis, tumor recurrence, the dose limiting side effects for chemotherapy and occurrence of drug resistance [9,10]. Thus, it is imperative to search for alternative treatment to conventional chemotherapy in order to improve overall outcomes for GC patients or to administer natural products in combination with classical chemotherapeutics to overcome cancer cell drug resistance problems. In recent years, an increasing interest in traditional herbal medicine research has been observed. Nowadays, numerous bioactive compounds have shown great valuable biological, pharmacological, and medicinal properties, including their potential anticancer activities.

In the light of these findings, a summary of research reports describing the results from studies on phyto-compounds, their low toxicity against normal cells, and their mechanisms of action in gastric cancer is considered by the authors to be of great significance in cancer prevention and therapy. Rationale for studies on natural products is the fact that some phytochemicals are being successfully used in clinical practice [11]. Moreover, the aim of the present review is to encourage researchers to screen biocompounds efficacy as potential new anticancer drugs. In this review, the authors have drawn great attention to isolated single metabolites and some total plant extracts. Until now, their ability to suppress the growth of gastric cancer cells is supported by many studies performed in vitro, and different mechanisms of its action have been proposed (Table 1). Here below, we present several groups of plant derived secondary metabolites (sulphur-containing compounds, polyphenols, alkaloids, terpenes) and characterize the effects they show in relation to gastric cancer treatment.

## 2. Biomarkers of Gastric Cancer

**Figure 1:**
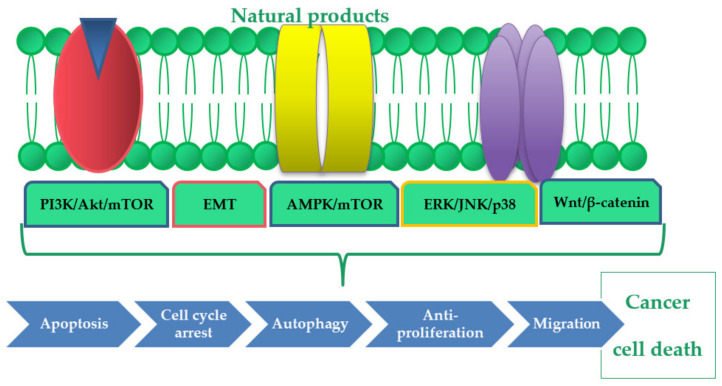
The molecular effects of natural products in gastric cancer cells.

Natural products are proved to exhibit several mechanisms of cancer-cells growth inhibition. Among them, induction of apoptosis, necrosis, autophagy, and cell cycle arrest have been listed by the researchers. These final anticancer effects that are triggered by plant metabolites can be achieved by a direct activation of several signaling pathways and by the reversion of cancer cells’ resistance. Table 1 and Figure 1 list the most important molecular effects caused by the compounds of natural origin. 

Gastric cancer cells express a wide variety of growth factors, hormones, and cytokines such as vascular endothelial growth factor (VEGF), interleukin-8 (IL-8), basic fibroblast growth factor (bFGF), or platelet-derived endothelial cell growth factor (PD–ECGF). The secretion of mediators varies depending on the histological type of gastric cancer cells. In the intestinal cancer type, the family of fibroblast growth factors (FGFs) is strongly expressed, including the EGF (epidermal growth factor), TGF-α (transforming factor α), EGF-CFC (epidermal growth factor-CFC), and AR (amphiregulin). However, in the diffusive type, the expression of TGF-β, IGF II (insulin-like factor II), and basic fibroblast growth factor prevail. EGF-CFC overexpression is associated with intestinal metaplasia [12]. TGF-β is known for its induction of the epithelial–mesenchymal transition (EMT), which plays an important role in the development and progression of malignancy of various human tumors [13].

Gastric cancer cells also produce neutrophilin-1 (NRP-1) and interleukin 1-α. NRP-1 is a coreceptor for the VEGF2 receptor on endothelial cells. EGF induces both expression of NRP-1 and VEGF, suggesting that the regulation of NRP-1 expression in gastric cancer is closely related to the EGF/EGFR system [14]. Interleukin 1-α acts as an autocrine growth factor for cancer cells and is an important factor in the expression of EGF and the EGF receptor. The interaction between interleukin 1 α (IL-1α) and the EGF/receptor system stimulates the development of gastric cancer [14]. A similar effect on gastric cancer is demonstrated by interleukin 6 (IL-6). Its concentration is elevated in the serum of patients with stomach cancer and it can be a prognostic factor on the stage of tumor development [15].

Additionally, IL-8, a member of the CXC chemokine family, plays an important role in the cancerogenesis of the stomach. IL-8, like vascular endothelial growth factor (VEGF) and basal fibroblast growth factor (bFGF), are produced by tumor cells and cause neovascularization in gastric cancer tissue [14].

NF-κB (nuclear factor kappa-light-chain-enhancer of activated B cells) is a ubiquitous transcription factor that is activated in many different tumors and plays a key role in tumor formation [16]. NF-κB pathway is involved in cell transformation, proliferation, induction of apoptosis, and angiogenesis. In gastric cancer cells, NF-κB pathway has been shown to be constitutively activated, and dysregulation of its component is used as a prognostic parameter in gastric cancer [17].

## 3. Phytocompounds with Potential Anticancer Activity on Gastric Cancer

The section lists primary and secondary metabolites of natural origin and characterizes the triggered molecular effects in gastric cancer cell lines. The extracts and single metabolites were selected from the Scopus database search for ‘gastric cancer’ and ‘natural products’ keywords that resulted in 302 records. The metabolites described below appeared in the newest publications, represent different groups of metabolites, and were thoroughly investigated towards their mechanisms of action. It is worth a note that recent 5 years bring a vivid development of phytotherapy of gastric cancer. Around 30 manuscripts were published each year within the last 5 years, whereas the previous decade delivered only ca. 10 records per year. The increase in publication records that stay within the scope of the discussed topics shows the topicality of the problem and an increasing importance of gastric cancer studies.

### 3.1. Primary Metabolites

#### *Cordyceps* *cicadae*

*Cordyceps cicadae* is a type of fungus from the Cordycipitaceae family often used in natural Chinese medicine due to its numerous healing properties. The fungus has been used to protect kidney function, improve vision [68], and prevent from cancer. Its fruiting bodies and corpus contain a variety of amino acids with lysine, glutamic acid, proline, and threonine as the most abundant ones, and unsaturated fatty acids with linoleic acid as the leading one that constitute 70% of the total fatty acids content [69]. Cordycepin (adenosine derivative), cordycepic acid (D-mannitol), and ergothioneine (a thiourea derivative of histidine) were proved to be the active components of its extracts [70]. The effects of alcohol extract from *Cordyceps cicadae* on gastric cancer cell lines SGC-7901 have been demonstrated in the study of Xie and collaborators. The results showed an inhibitory effect of the extract on the proliferation of SGC-7901 cancer cells by increasing cellular stress and cell cycle arrest at S phase. The IC_50_ value of the tested extract was found to be 121.4 μg/mL and may be influenced by the presence of the major extract’s metabolite: cordycepin [71]. In addition, the apoptosis induction by concomitant upregulation of cleaved-caspase 3, -9, and –PARP (Poly (ADP-ribose) polymerase), and downregulation of survivin was described. Cordycepin has also been shown to attenuate the proliferation of MGC-803, SGC-7901, and HGC-27 gastric cancer cells by targeting the PI3K/Akt pathway involved in tumor initiation and progression [18]. The spread of cancer cells from the primary tumor to another part of the body is still a clinical challenge. Pooled data indicate that the majority of cancer deaths are caused by metastases [72] and its initiation has been associated with changed adhesion ability between cells and the extracellular matrix (ECM), damaged intercellular interaction and the ECM degradation [73] as well as epithelial–mesenchymal transition (EMT) pathway and overexpression of matrix metalloproteinases (MMPs) [74]. EMT is a physiological process in which epithelial cells lose their adhesion property and acquire cell phenotype characterized by the increased expression of mesenchymal genes [75]. Moreover, cordycepin was found to inhibit the gastric cancer cells metastasis by activating an expression of epithelial marker such as E-cadherin and inhibiting mesenchymal marker such as Vimentin and E-cadherin repressors, including Snail and Slug [18].

### 3.2. Secondary Metabolites

#### 3.2.1. Sulphur-Containing Compounds

##### *Allium* *sativum*

*Allium sativum* (garlic), from the Amaryllidaceae family, is known for its numerous medicinal properties [76] and has been selected as a representative of well-studied sulphur-containing compounds. Its main active natural constituent such as alliin is a precursor of allicin (diallylthiosulfinate) that is formed in enzymatic reaction catalyzed by alliinase which is released upon cutting, crushing or chewing of the *Allium* vegetables [77]. The transformation of sulphur-containing compounds in the air with the participation of water observed in the case of garlic is typical for glucosinolates and necessary for induction of their biological activity. Allicin (Table 2) is biologically the most active compound of garlic and under the action of certain enzymes in gastric juice it is degraded to various organosulphur compounds, including diallyl sulfide (DAS), diallyl disulfide (DADS), diallyltrisulfide (DATS), dithiines, and ajoene [78]. It has been found to exhibit a wide range of biological and pharmacological activities, including antimicrobial, antifungal, and antioxidant properties, cardiovascular diseases risk reduction, immune function improvement, hypoglycemic and hypocholesteremic effects [73]. In 1983, Belman et al. [79] first noted the potent anticancer activity of garlic oils. Allicin has been reported to inhibit the growth of gastric cancer cell lines mainly through inducing cell cycle arrest at G2/M phase and apoptosis [19]. In vitro studies on human gastric cancer cell line SGC-7901 using *Allium sativum* extracts with 3, 6, 12 mg/L of allicin demonstrated that already 3mg/L of allicin exhibited a proapoptotic effect and inhibited the viability of cancer cells. At the molecular level, both caspase-dependent mitochondrial and death receptor apoptotic pathway occur in SGC-7901 cells following allicin treatment. Another mechanism by which allicin can induce apoptosis in gastric cancer cells is decreasing the activity of telomerase that is important in context of successful cell division [20]. Enhanced expression of cleaved caspase 3, altered expression levels of apoptosis-associated proteins such as Bcl-2 and Bax and consequently promotion of apoptosis has also been observed in MGC-803 and BGC-823 human gastric carcinoma cell lines in response to increased concentrations of allicin. The underlying mechanism of the proapoptotic effects of allicin in MGC-803 may involve the activation of the intracellular p38 mitogen-activated protein kinase (MAPK) signal transduction pathway that is often accompanied by activation of caspase-3 [21]. In 2005, Chinese scientists published the results of their research on the medicinal properties of garlic which were performed in 878 patients from Shanghai and Quingdao with the help of a standard questionnaire. Their research showed a mutual relationship between regular consumption of garlic and onions, and a reduction in the incidence of stomach cancer. In addition, along with an increase in the dose of administered garlic, the risk of stomach cancer located in its distal part decreased [80]. A systematic review and meta-analysis also confirmed the effect of garlic intake in high doses on decrease of gastric cancer mortality [81].

*Helicobacter pylori* is considered as a key factor in development of gastric cancer. A number of mechanisms have been suggested to explain the inhibitory effects of allicin on the growth of *Helicobacter pylori* bacteria and anti-inflammatory effects of this biocompound. One of them is the ability of allicin to react with the sulfhydryl group (SH) of proteins located on the surface of *H. pylori*, including heat shock proteins (HSPs), urease as a major virulence factor, and lipopolysaccharidase enzyme which induce the production of inflammatory factors such as CRP, IL-8, and TNF-α [82]. In addition, allicin prevents the activation of NF-κB-dependent TLR (Toll-like receptor) signaling pathway that plays critical role in inflammation and cancer. Studies revealed that allicin can bind with the cysteine in the extracellular and cytoplasmic domains of TLR receptors and finally blocks induction of immune responses [83].

#### 3.2.2. Polyphenols

Polyphenols are a diverse group of plant metabolites with high polarity due to the presence of hydroxyl groups and unsaturated benzene rings in their structure. Among them, several types of natural products can be listed: from simple compounds such as phenolic acids or naphthoquinones, medium-sized molecules like flavonoids, to large-molecule metabolites such as tannins. All of the above-mentioned groups have a confirmed antitumor effect in gastric cancer.

##### *Camellia* *sinensis*

*Camellia sinensis* is an evergreen shrub from the tea family, which is commonly used for the preparation of the most popular drink in the world such as tea. Its broad therapeutical properties have been studied for many years and denoted an association of its consumption with a reduction in the risk of gastric cancer [84]. Beneficial properties of green tea result from the high content of polyphenols in its infusions. The anticancer action of the main polyphenol found in green tea, epigallocatechin-3-gallate (EGCG), has been associated with its pro- and antioxidant activities. EGCG and the other catechins present in tea extracts are also widespread in numerous plant species. That is why the studies on their impact on gastric cancer cells is of high significance. Recent studies revealed that EGCG may trigger radical oxygen species (ROS) generation and inhibit angiogenesis process through ROS-mediated activation of AMP-activated protein kinase (AMPK). As a result, AMPK downregulates mTOR signaling pathway leading to decrease in the level of VEGF in cancer cells [22]. Hypoxia-inducible factor 1 (HIF-1α) and its downstream target VEGF play a critical role in tumor growth, angiogenesis, and metastasis and their expression at protein level were effectively inhibited in gastric cancer SGC-7901 cells following treatment with EGCG [23]. Meanwhile, EGCG induces apoptosis by scavenging activity of ROS that have been proved to stimulate gene expression of antiapoptotic protein B-cell lymphoma-2 (Bcl-2) via activation of NF-κB factor [85]. The other mechanisms of apoptosis induced by EGCG in cancer cells may involve the activation of the apoptosis-related proteins, such as caspase-3, caspase-9 and PARP [24], Bax and cytochrome C (cyt C) [25]. Moreover, antitumor potency of EGCG was also shown to result from its ability to suppress growth factor receptors such as epidermal growth factor receptor (EGFR) [26] and insulin-like growth factor receptor (IGF-1R) that play significant roles in tumor survival and growth. It has been proven that EGCG effectively reduced activity of both receptors and of their downstream signaling molecules such as extracellular signal-regulated kinase (ERK1/2), cyclin D1, and kinase Akt [86]. Modulation of molecular pathways by EGCG also affects transcription factors such as Signal Transducer and Activator of Transcription 3 (STAT3) and Activating Protein-1 (AP-1) implicated in pathogenesis of cancer. Wnt signaling pathway regulates different cellular processes, including cell fate, movement, polarity, and organogenesis. Altered Wnt pathway has been implicated in several events, including gastric carcinogenesis [87]. Recent work revealed that β-catenin, a crucial Wnt signaling transcription factor is overexpressed in gastric cancer. EGCG significantly decreased the expression of phosphorylated (p)-β-catenin (Ser552) in SGC-7901 gastric cancer cells by downregulating expression of phosphorylated (p)-GSK3β (Ser9). Phosphorylation of β-catenin at Ser552 was associated with its cytoplasmic accumulation, nuclear translocation, transcription of its downstream genes, including *ccnd1*, *c-myc*, and *c-jun* and cell proliferation induction [27]. EGCG is also an active inhibitor of DNA methyltransferase (DNMT) and histone deacetylases (HDAC), which aberrant expression of is frequently observed in various human cancers and may lead to gene silencing, in particular tumor suppressor genes [28,29]. These data and meta-analysis of observational studies allow the conclusion that long-term and high-dose consumption of green tea may reduce the risk of gastric cancer [88]. 

Several clinical trials conducted in China on different groups of patients drinking green tea confirmed its anticancer properties. These valuable tests also apply to the occurrence of gastric cancer. In order to gather information on possible stomach cancer risk factors and to assess the role of green tea consumption, a personal questionnaire was used. It has been shown that regular drinking of tea (≥35 g/week) significantly reduced the risk of gastric cancer incidence. It was also proved that lower temperature of consumed tea and longer intervals between filling a cup of tea and drinking were beneficial [88].

##### *Cardiospermum* *halicacabum*

*Cardiospermum halicacabum* is a woody delicate climbing plant belonging to the Sapindaceae family. The alcohol extracts of this plant are used traditionally to treat eczema, psoriasis, lupus, and diabetes. It also exhibits digestive effects and has been commonly used in peptic ulcer disease and duodenitis [89]. The influence of the aqueous *C. halicacabum* extracts rich in polyphenols and tannins on gastric cancer cells AGS, SNU-5, and SNU-16 were proved in a study joining the application of the plant together with gold nanoparticles. Biosynthesized *C. halicacabum*–gold nanoparticles (CH-AuNP) showed the highest activity against AGS cells with the IC_50_ value of 25 μg/mL. Molecular mechanism of the antitumor activity of CH-AuNP in AGS cancer cells was associated with proapoptotic cell death initiation mediated by an increase in the intracellular production of ROS. Nanoparticle CH-AuNP marked an increase in the expression of proapoptotic proteins (Bax, caspase-3 and caspase-9) and a decrease in the expression of antiapoptotic proteins (Bcl-xl and Bcl-2) [30].

##### *Plumbago* *zeylanica*

*Plumbago zeylanica* (Plumbaginaceae) is naturally spread in India, where it is used as an antimicrobial, antiatherosclerotic, and anticancer agent. The major constituent of its root extracts was plumbagin, a naphthoquinone derivative (5-hydroxy-2-methyl-1,4-naphthoquinone) (Table 2). Antiproliferative effects of plumbagin have been reported in both in vitro and in vivo studies against various malignances such as breast, pancreatic, lung, prostate, melanoma cancers, and leukemia [90]. Previous studies clearly demonstrated anticancer effects of plumbagin in various types of gastric cancer cell lines, including AGS, SGC-7901, and MKN-28 which were found to be due to the induction of apoptotic cell death either through suppression of Akt and STAT3 phosphorylation [31] or inhibition of NF-κB signaling pathway [32]. NF-κB is a family of transcription factors, among them the most common is the heterodimer of RelA/p50 (p65/p50). In most types of the normal cells under resting state, NF-κB factor is present in an inactive form by binding to its IκBα inhibitory protein. The activation of cell membrane receptors by different stimuli leads to the phosphorylation of IκBα kinase protein (IKKβ) and in turn phosphorylation and degradation of IκBα inhibitor by proteasome. The released NF-κB dimer is translocated from cytoplasm to nucleus where it binds to DNA and regulates the expression of targeted genes such as inhibitor of apoptosis 1 (IAP1), X-linked inhibitor of apoptosis (XIAP), Bcl-2, Bcl-xl, and VEGF [91]. Plumganin was demonstrated to reduce proliferation and survival of gastric cancer cells by modulating phosphorylation of NF-κB pathway proteins and downregulation of NF-κB-related gene products. It was observed that cell viability of SGC-7901, MKN-28, and AGS cells after the administration of plumbagin was inhibited in a dose-dependent manner with IC_50_ values of 3.594, 2.564, and 1.903 μg/mL, respectively [32]. Furthermore, plumbagin induces autophagic cell death and remarkably inhibits both the migration and invasion of AGS cells at IC_50_ of 1.504 μg/mL, considered as imperative for cancer cell metastasis [92].

##### *Chrysosplenium* *nudicaule*

Spearmary (*Chrysosplenium*) is a representative of the Saxifragaceae botanical family naturally occurring in Japan and China, where it is the most abundantly represented by different varieties. First data confirming the cytotoxic and antitumor activities of *Chrysosplenium nudicaule* extract originates from the year of 2000 [93]. Two main active flavonoids isolated from its extracts: 6,7,3′-trimethoxy-3,5,4′-trihydroxy-flavone (TTF) and 5,4′-dihydroxy-3,6,3′-trimethoxy-flavone 7-*O*-*β*-d-glucoside (DTFG), inhibited cancer cell growth of human K562 leukemia cells [94] and SGC-7901 gastric cancer cells [34]. The results showed that gastric cancer cells were more sensitive to the plant’s ethanol extracts containing 32, 16, 8, 4, and 2 μg/mL TTF than DTFG. TTF inhibited SGC-7901 cell growth with IC_50_ value of 8.33 μg/mL. Further studies also revealed that TTF was able to block the cell cycle progression and induce apoptosis in SGC-7901 cells. Proposed mechanism of apoptotic cell death induction in SGC-7901 cells treated with TFF includes the downregulation of Bcl-2 expression and the upregulation of Fas expression. 

#### 3.2.3. Terpenes

Terpenes constitute one of the largest groups of secondary metabolites that includes fragrant low polarity scaffoldings—the derivatives of isoprene—that are characterized by a wide range of pharmacological properties including the anticancer activity demonstrated by different mechanisms of action.

##### *Saussurea* *lappa*

*Saussurea lappa* (Asteraceae) root has been successfully used in gastrointestinal ailments. The plant extracts rich in costunolide—a sesquiterpene lactone—were proved to exhibit anticancer and narcotic properties [95], however, its mechanism of action has not been fully elucidated. The anticancer potential of costunolide against gastric cancer has been determined on the following cell lines AGS, BGC-823. Studies on AGS cells found that *S. lappa* resulted in apoptosis and cell cycle arrest at G2 phase in a dose- and time-dependent manner through p53 and p21Waf1 proteins induction and concomitant reduction of cyclin B1. Furthermore, the treatment of AGS cells with the plant extract led to the activation of proapoptotic molecules including Bax and caspase-3, and suppression of antiapoptotic Bcl-2 [35,36]. Based on the above data, it can be concluded that effects of *S. lappa* terpene on the gastric cancer cells growth are associated with its influence on the expression of genes controlling cell cycle and apoptosis. Significant alterations in mitochondrial membrane potential (ΔΨm), closely related to induction of mitochondrial-mediated apoptotic pathway, has been observed in BGC-823 gastric cancer cells treated with costunolide. Simultaneously, changes in expression of apoptosis related proteins and genes were detected. An increased expression of proapoptotic proteins (Bax, Bak), cleaved caspase 9, -7, -3, and cleaved poly ADP ribose polymerase (PARP) proteins and decreased expression of the antiapoptosis protein such as Bcl-2 have been shown [37]. Similar mechanism of induction of apoptosis has also been found in SGC-7901 cell line [38]. Other study demonstrated also that costunolide significantly reduced vascular endothelial growth factor (VEGF) secretion and its mRNA levels in AGS cell line. The binding of VEGF with its receptors (VEGFR) on vascular endothelial cells promotes formation of new blood vessels. This process is called angiogenesis and has an important aspect in tumor invasion and metastasis [39].

##### *Nigella* *sativa*

*Nigella sativa*, or black cumin, is a plant from the Ranunculaceae family, known for its medical properties related to the activity of the digestive system, including the antidiabetic action. Its extracts are rich sources of terpenes, among which thymoquinone, γ-terpinene, thymol, and β-caryophyllene were identified as the most abundant volatiles [96]. It has been shown that the plant extract has the ability to lower the oxidative stress and maintain the integrity of pancreatic cells. Black cumin also shows beneficial effects on regeneration and proliferation of pancreatic β-cells, improving glucose metabolism, increasing insulin secretion, and reduction of the intestinal glucose absorption [97]. Its therapeutical properties are associated with the presence of thymoquinone (TQ) in plant extracts, which has been reported to exhibit multiple therapeutic applications such as anticancer, anti-inflammatory, antioxidant, and antimodulatory. TQ inhibits the progression of various types of cancers including leukemia, breast adenocarcinoma, colorectal, pancreatic, prostate, and hepatic cancer [42,43]. Lei et al. for the first time reported the anticancer activity of TQ against BGC-823, SGC-7901, MGC-803, and HGC-27 gastric cancer cells. It was found that TQ can induce apoptosis in studied gastric cancer cell lines in a dose- and time-dependent manner. Furthermore, TQ has been shown to be able to sensitize gastric cancer cells to conventional chemotherapy such as 5-fluorouracil (5-FU) and cisplatin (CDDP) both in vitro and in vivo. Studies have demonstrated that the combined treatment of TQ with 5-FU or CDDP significantly augments chemotherapeutic-induced antitumor effects on gastric cancer by enhancing the activation of the mitochondrial apoptotic pathway and some of the key proteins of this pathway, including Bax, Cyt c, AIF, cleaved caspase-9, and -3 [40,41]. Cancer multidrug resistance (MDR) is defined as the ability of cancer cells to gain resistance to both conventional and novel chemotherapy agents and is one of the major challenges in clinical practice [98]. One of the mechanisms contributing to MDR is overexpression of P-gp, ATP-dependent drug efflux membrane transporter, and its inhibition has been suggested as a potential strategy to overcome drug resistance in cancer [99]. It has been shown that TQ exerted its chemotherapy enhancing effect through downregulation of P-gp mediated by increase in the levels of phosphatase and tensin homolog (PTEN) and then decrease in the levels of p-Akt (S473) and p-Akt (T308) in gastric cancer cells [42,43]. In fact, PTEN is a tumor suppressor protein and inhibits the activation of phosphoinositide 3-kinase (PI3K/Akt) cascade that plays a very important role in both the progression of gastric cancer and also acquisition of the chemoresistance properties. The loss of PTEN activity has been associated with abnormal cell growth and apoptosis escape [100]. TQ also inhibited cell migration ability of MGC-803 and SGC-7901 gastric cancer cells by Akt-dependent downregulation of the expression of mesenchymal genes (*N-cadherin*, *Vimentin*, and *Twist*) and upregulation of the expression of epithelial genes such as *E-cadherin* and *cytokeratin-19* [42]. Moreover, TQ has been reported to suppress the expression of STAT3, a member of the STAT protein family, playing a critical role in the Janus kinase (JAK)/STAT signaling pathway. A few years ago, STAT-3 was considered an acute phase response element having several cellular functions such as inflammation, cell survival, invasion, metastasis and proliferation, genetic alteration, and angiogenesis [101]. Abnormal expression or constitutive activation of the JAK/STAT pathway component were found to be in a variety of cancers such as gastric, colon, breast, and lung. The studies of Zhu et al. [44] revealed that the mechanism of TQ-mediated STAT3 inhibition in HGC27 gastric cancer cells involved the inhibition of upstream protein kinases (JAK and Src), and presumably the suppression of STAT3 phosphorylation at Tyr705. The phosphorylation induces STAT protein dimerization, which is required for its nuclear translocation, DNA binding, and transcriptional activation of target genes [102]. TQ also suppressed the expression of several STAT-3 regulated genes, including proliferative (cyclin D1), antiapoptotic (Bcl-2, survivin), and angiogenic (VEGF) gene products in HGC-27 [44]. Lowering *VEGF* gene expression and induction of apoptosis were also observed in AGS gastric cells treated with TQ [45]

##### *Euphorbia* *lunulata*

*Euphorbia lunulata* is a perennial herb from the Euphorbiaceae family commonly spread around Eurasia. Its stems are rich in white milk juice which is attributed to the healing properties of the species. The composition of the above-mentioned milk juice was analyzed and the presence of the following diterpenoids: ingenol 3,20-dibenzoate, 3,16-dibenzoyloxy-20-deoxyingenol, and 3,13,16-tribenzoyloxy-20-deoxyingenol was reported [103,104]. These constituents were found to inhibit cell proliferation and induce apoptosis in vitro in the MCF-7 and NCI-H460 cell lines [105,106]. *E. lunulata* extract was shown to exhibit significant inhibitory effects on lung, cervical, gastric, breast, and liver cancers, and its anticancer effects are mainly manifested in cell cycle arrest, inhibition of cell apoptosis, and decrease in the migration ability of cancer cells [107]. The anticancer effects of *E. lunulata* extract have also been studied on adriamycin resistant strain of human gastric carcinoma SGC-7901 (SGC-7901/ADR). *E. lunculata* extract, obtained from a fresh aerial part of the plant in ethanol and then in n-hexane, successfully inhibited the proliferation, migration and invasion of SGC7901/ADR cells at the concentration range of 2.5–80 μg/mL.

The cell cycle block at the G2/M phase and cell apoptosis induction has been observed. It was associated with increased activities of caspase-3, -8, and -9, the apoptosis-promoting protein Bax and decreased expression of the apoptosis-inhibiting protein Bcl-2 [46].

##### *Euphorbia* *esula*

Another *Euphorbia* species has been reported to exhibit marked anticancer properties against gastric cancer. *Euphorbia esula* (Euphorbiaceae), the lanceolate spurge is a perennial milk-containing plant traditionally used in the treatment of skin warts in traditional Chinese medicine. Its effectiveness in the anticancer activity on gastric cell lines was confirmed by Fu and colleagues [47]. The anticancer activity of the water extracts from the fresh aerial parts of the plant harvested in the Yanan mountain area was studied on human gastric cancer cells SGC-7901. The study showed that *E. esula* extract inhibited proliferation and induced apoptosis in SGC-7901 cells. The effect was caspase-dependent and included increased *Bax* expression and downregulation of *Bcl-2* gene [47]. These results suggest that *E. esula* latex extract induces apoptosis of SGC-7901 cells by external signaling or a membrane-dependent pathway.

##### *Dioscorea* *bulbifera*

The onion shed (*Dioscorea bulbifera*) is a perennial vine with tuberous rhizomes which belongs to the family of Dioscoreaceae. The plant is known for its medicinal properties and has been used in the pharmaceutical industry for the synthesis of steroid hormones. Its preparations have been traditionally recommended for rheumatism, constipation, poisoning, underdevelopment of the breast glands, premenstrual syndrome, excessive menstrual bleeding, sperm whalebone, or respiratory tract diseases. Recent studies shed new light on its application as they confirmed a strong effect of the alcohol extracts of the plant on gastric cancer cells, when applied in the concentration range of 0.1–0.8 mg/mL. The inhibitory effect of the extract on the proliferation of human gastric cancer cell line SGC-7901 has been demonstrated. With increasing concentration of the extract, an increased rate of inhibition of cell division was observed [48]. The active antitumor substance derived from the extract of this plant is considered to be diosbulbin B from the class of naphtofurans or diterpene lactones, which was isolated from its extracts by high-performance liquid chromatography (HPLC) for pharmacological tests [108].

#### 3.2.4. Alkaloids

Secondary metabolites of plant or animal origin bearing a nitrogen atom in its structure (in a heterocyclic ring, side chain or a nitro group) are called alkaloids. This diverse group of compounds that gathers ca. 27,000 compounds is biogenetically derived from various amino acids which determine their type [109]. Among several major groups of alkaloids, the derivatives of isoquinoline are the richest in representatives. Nevertheless, this group of metabolites belongs to the most active components of plant extracts in terms of pharmacological effects on a human body. Here below, we present some examples of a different chemical type together with the differing mechanisms of action against gastric cancer cells.

##### *Coptis* *chinensis*

The roots and the root bark of *Coptis chinensis* (Ranunculaceae) have become the flagship sources of several types of isoquinoline alkaloids. Their extracts were proved to contain berberine, palmatine, coptisine, columbamine, jatrorrhizine-protoberberine derivatives, together with other aporphine and benzylisoquinoline alkaloids present in smaller quantities [110]. These thin-rhizomed perennials have been used for centuries as digestive, antiprotozoan, anti-inflammatory, and antibacterial remedies in the traditional medicine of Asia [111]. Several studies confirm a marked anticancer potential of the protoberberines in the therapy of gastric cancer. Among the above listed alkaloids, berberine is certainly the best studied metabolite of Ranunculaceae and Berberidaceae representatives. Its high content in different plant species, simple isolation protocols, and low toxicity [112] favor its high availability. Berberine (BBR) was also found to exert its antitumor effects against gastric cancer in both in vitro and in vivo studies. According to Zhang and collaborators [49] BBR suppressed the growth of BGC-823 gastric cancer cells with the IC_50_ value of 8.12 μg/mL. It has been shown that BBR induced the autophagic cell death through inhibiting ERK/JNK/p38 MAPK/the mammalian target of rapamycin (mTOR)/p70 ribosomal S6 protein kinase (p70S6K) and PI3K/Akt signaling pathways in BGC-823 cells. Instead, the involvement of Akt/mTOR/p70S6K/S6 pathway in BBR-mediated inhibition of cell proliferation and induction of cell death was described by Yi at al. [50]. BBR increased the expression levels of cleaved PAPR and caspase-3, and impaired mitochondrial membrane potential (Δψm) in BGC-823 and SGC-7901 gastric cancer cells. Slowed proliferation of AGS and SGC-7901 gastric cancer cells was also associated with downregulation of the proto-oncogene c-myc expression, cell cycle arrest at G0/G1 phase with the decreased expression of cyclin D1, whereas the antimetastatic potential of BBR on gastric cancer cell lines was manifested through downregulating MMP3 [51]. It was reported that the expression level of MMP-3, known as inducers of EMT, was negatively correlated with gastric cancer development [113,114,115]. Moreover, the molecular mechanism for anticancer effects of BBR was related with its ability to downregulate the expression of Hepatocyte Nuclear Factor 4α (HNF4α) and its downstream target genes through increasing the protein level of phospho (p)-AMPK [51]. The changes in HNF4α gene expression have been associated with many types of cancers, including gastric adenocarcinoma [116]. AMPK mediated the phosphorylation of HNF4α decreased significantly the protein and mRNA expression levels of WNT5A, cytoplasmic β-catenin and cyclin D1 in vitro, and increased significantly the expression of E-cadherin [51] thus indicating probably a cross-talk between the AMPK metabolic pathway and the WNT signaling pathway [117]. The studies of Wang and colleagues [52] showed a marked inhibitory effect of BBR on SNU-1 gastric cancer cells at a similar concentration as in the previously described studies (the IC_50_ of 10.1 μg/mL). BBR is reported to promote apoptosis in SNU-1 cells by activating caspases and the enhancement of the Bax/Bcl-2 ratio. These effects could be due to the influence of the alkaloid on the p38/JNK MAPK and NF-κB pathways. Interestingly, BBR in combination with standard chemotherapy can achieve better anticancer effects [53]. A synergistic inhibition of survivin and STAT3 level resulting in enhanced cell death were observed in AGS gastric cancer cells after treatment with BBR with 5-FU [54]. BBR has also been shown to overcome cisplatin resistance in SGC-7901/DDP and BGC-823/DDP gastric cancer cells and induce caspase dependent apoptosis through restoring microRNA (miR)-203 expression [118]. It belongs to a family of non-coding, small RNA molecules that functions to post-transcriptionally regulate gene expression. [119]. You et al. findings revealed that Bcl-w transcript coding of an antiapoptotic protein was negatively regulated by miR-203 [118].

Epiberberine (EPI), another alkaloid isolated from *C. chinensis*, has also been shown to exert its anticancer properties in gastric cancer cells. The effects of EPI action were studied on two gastric cancer cell lines [120], of which MKN-45 (harboring wild-type p53) was more sensitive to alkaloid than HGC-27 (harboring mutant p53). It was established that EPI inhibited the growth of MKN-45 cells in a dose-dependent manner through both cell cycle arresting and targeting p53-dependent mitochondrial-associated apoptotic pathway. In response to a stress signal, wild-type tumor suppressor protein p53 translocates to mitochondria where it may interact with various pro- and antiapoptotic Bcl-2 family proteins governing the integrity of the mitochondrial membrane. Upon activation, the oligomerization of proapoptotic Bax and Bak proteins onto the mitochondrial membrane leads to the formation of pores and then apoptogenic factors being released [121]. The analysis of the expression of p53 pathway related proteins in MKN-45 cells treated with EPI revealed markedly an increase in the expression of p53, p21, p27, and the ratio of Bax/Bcl-2. The expression of cytochrome C and cleaved caspase-3 were also significantly increased, while that of XIAP was decreased after EPI treatment. ROS generation concomitant with decreased mitochondrial membrane potential (ΔΨm) in MKN-45 gastric cancer cells were found to be also related in EPI induced apoptosis [120].

##### *Stephania* *tetrandra*

The root of *Stephania tetrandra* (Menispermaceae), a perennial herbaceous vine spread in Taiwan and China, is a source of a promising drug candidate—a bisbenzylisoquinoline alkaloid: tetrandrine (TET). The presence of this compound was also confirmed in other species, e.g., in the extracts from the representatives of *Macleaya*, *Mahonia*, *Cyclea*, *Cocculus*, and *Cissampelos* genera [122]. Tetrandrine has been traditionally used as a diuretic and antihypertensive drug. Recent findings shed a new light on its pharmacological application and underline the marked cancer growth inhibitory properties of this alkaloid [123]. Tetrandrine-mediated anticancer abilities have been reported toward the following gastric cancer cell lines HCG-27 and BGC-823 with defined IC_50_ values of 16.1 and 4.471 μg/mL, respectively. In previous studies, it has been shown that TET can efficiently enhance the cytotoxicity of chemotherapeutic agents such as paclitaxel (Ptx), 5-FU, oxaliplatin (Oxa), and docetaxel (Doc) used for the treatment of gastric cancer [33,55]. Studies of Bai et al. [56] and Qin et al. [57] revealed that the potential molecular mechanisms corresponding to the antiproliferative effects of TET in gastric cancer cells seem to be relating to apoptosis and autophagy initiation. The mitochondrial intrinsic pathway of apoptosis induced by TET in both BGC-823 and HGC-27 gastric cancer cells was depending on the activities of proteins of the Bcl2 family and caspase cascade pathway. An increased Bax/Bcl2 ratio caused mitochondrial disfunction and finally the activation of caspase-3. In both studies on gastric cancer cell lines, cytochrome C release and Apoptotic protease activating factor 1 (Apaf-1) upregulation were observed. The treatment of HGC-27 gastric cancer cells with TET resulted in a significant increase in the expression of proteins engaged during autophagy initiation and autophagosome formation, such as LC3-II and Beclin-1 [56]. Several studies showed that PI3K/Akt/mTOR pathway played a negative role in regulating autophagy [124]. Studies of Bai et al. [56] showed that TET-induced autophagy in gastric cancer cells has a connection with dose-dependent inhibition of Akt phosphorylation and its downstream signaling proteins such as mTOR, p70S6K, and the eukaryotic initiated factor 4E-binding protein 1 (P-4EBP1).

##### *Piper* *longum*

The roots of a flowering wine, *Piper longum* (known as long pepper, Piperaceae) traditionally used in Ayurvedic medicine, are sources of an amide alkaloid with pungent taste—piperlongumine (PL) that has proved to be effective against various ailments including cancer, neurogenerative disease, arthritis, melanogenesis, lupus nephritis, and hyperlipidemic [125]. The treatment of gastric cancer cells with PL alone or in combination with Oxa significantly resulted in the accumulation of intracellular ROS, p38/JNK signaling pathway activation and thereby the induction of cell apoptosis. It has been found that PL induced SGC-7901 and BGC-823 gastric cancer cells growth inhibition by suppressing the activity of thioredoxin reductase 1 enzyme (TrxR1) [58,59] which regulates cellular redox balances and is a major line of cellular defense against ROS. [126]. Interestingly, PL inhibited AGS and HGC-27 gastric cancer cells proliferation by increasing GADD45α protein levels, which led to G2/M phase arrest [60]. Mitotic entry was shown that requires the activities of Cdc2 or cyclin-dependent kinase 1 (Cdk1) which are positively regulated by association with cyclin B1 and negatively regulated by interacting with GADD45α protein [61]. Indeed, PL administration resulted in lowered both cyclin B1 and Cdc2 expression in AGS and HGC-27 cells and thus inhibition of cell cycle progression. In addition, the activation of cleavage of PARP and caspases, such as caspase-9, -7, -3, has been observed after the treatment of gastric cancer cells with PL. The studies of Daun et al. demonstrated that downregulation of XIAP, one of the inhibitors of caspases, may contribute to a powerful proapoptotic effect of PL and PL-induced apoptosis [60]. Furthermore, as shown in studies of Song et al. [62], PL inhibited the proliferation, cell cycle progression, as well as invasion and migration of MKN-45 and AGS gastric cancer cells through suppression of the JAK/STAT signaling pathway and downregulation of the expression of target genes of STAT-3, such as Ki-67 (a proliferation marker), cyclin D1 (a cell cycle regulator), MMP-9, and Twist (a EMT markers). Liu et al. indicated the role of the FOXO3A protein in PL-mediated cancer cell death [63]. The transcription factor forkhead box O 3A (FOXO3A) is a tumor suppressor that controls a variety of cellular processes including apoptosis, proliferation, cell cycle progression, DNA damage, and tumorigenesis [127]. The treatment of MGC-803 gastric cancer cells with PL induced nuclear translocation of FOXO3A and subsequently its binding to the BIM gene promoter. It resulted in the upregulation of the proapoptotic protein BIM expression and activation of intrinsic apoptosis. The proapoptotic activity of FOXO3A was associated with PL-mediated Akt inhibition [63]. It has been shown that Akt phoshorylates FOXO3A at Thr32, Ser235, and Ser315 and promotes its cytoplasmic retention [128].

##### *Sophora* spp.

*Sophora* is a genus of Fabaceae botanical family that comprises about 45 species of little trees or big shrubs widely distributed around the globe [129]. Among several species, *Sophora flavescens* and *Sophora japonica* are well studied and implemented in phytotherapy these days and were used in the treatment of dermatological and gynecological problems [65]. Their root extracts are rich sources of tetracyclic quinolizidine alkaloids like matrine, matrine oxide, sophocarpine (present also in *Oxytropis ochrocephala* extracts) [130], oxosophocarpine, sophoridine, and flavonoids from the groups of flavanones, pterocarpan flavonoids, and prenylflavonoids [131,132]. Alkaloids from the root extracts became of interest in terms of their anticancer properties. Around 20 scientific papers on the application of its alkaloids in chemoprevention were published within the last 10 years. The researchers focus on the study of all above-mentioned alkaloids, however, matrine (MA) and oxymatrine (OMA) seem to be good examples of the pharmacological activity profile of the Sophora alkaloids. 

The inhibitory effect of MA on gastric cancer cells growth has been emphasized in many in vitro studies. Different mechanisms underlying the antitumor action of MA in gastric cancer cells have been shown, including cell cycle arrest at G1 and G2/M phase, apoptosis induction either via increasing proapoptotic molecules of Bcl-2 family (the intrinsic pathway) or upregulation of Fas (CD95/APO-1)/Fas ligand (FasL) expression (the extrinsic pathway) and autophagy associated with upregulation of Beclin-1 expression. [64,65,66,133]. MA also significantly inhibited the cell migration and adhesion of BGC-823 gastric cancer cells by changing the subcellular distribution of the cytoskeleton protein vasodilator-stimulated phosphoprotein (VASP) and formation of actin stress fibers [67]. Peng et al. studies [134] revealed that MA inhibited proliferation and metastasis of SGC-7901 gastric cancer cells mainly by downregulating uPA expression through inactivation of the PI3K/Akt pathway. Moreover, the treatment of MKN-28 and SGC-7901 gastric cancer cells with MA caused inhibition in the proliferation and migration by suppressing miR-93-5p. The alterations in the expression of miR-93-5p positively regulate its target gene coding AHNAK protein that plays a pivotal role in the inhibition of various tumors. 

## 4. Future Perspectives: In Vivo Studies and Clinical Trials

Plant extracts are mixtures of metabolites characterized by a different chemical nature and structure. For years, these compounds have been treated as a scaffolding for semisynthetic or synthetic drugs, some of which are now used as first-line drugs in various diseases. The anticancer drugs are one example, where chemotherapeutic agents have been inspired by natural resources. As an example, docetaxel and carbasitaxel, the semi-synthetic derivatives of paclitaxel, isolated from the pacific yew (*Taxus brevifolia*), that are effective in the treatment of breast, ovarian, and lung cancer, or irinotecan and topotecan, semisynthetic anticancer derivatives of camptothecin used in the treatment of colorectal and rectum cancer should be listed.

The results of in vitro tests on cell lines described in this review are undoubtedly important, as they constitute the first screening stage of searching for new anticancer drug candidates. These studies allow to determine the toxicity of the tested substances in terms of tumor cell lines and normal cell lines, to observe their strength in relation to the currently used drugs and to determine their mechanisms of action. The purpose of this review was to compile the knowledge based on the in vitro tests in order to draw attention to new emerging plant substances that may be of importance in the treatment of gastric cancer in the future. With this review, the authors would also like to emphasize the importance of fractionation and the isolation of single molecules from plant extracts. Knowing the identity of individual constituents with high pharmacological potential is crucial in the process of drug delivery.

At this point, it is worth emphasizing that for some plant extracts in vivo studies on animals suffering from gastric cancer and clinical trials in humans are already being conducted. These data have been collected in the review article of Mao and co-workers [135]. The described studies bring numerous drug candidates closer to the final stage of testing, confirming their anticancer properties.

We would like to conclude this article by listing some important in vivo tests both in animals and in humans in the form of two tables (Table 3 and Table 4) which show the significance of natural products in the treatment of gastric cancer in living organisms. 

The results of these surveys that are still small in number in comparison with in vitro assays confirm the significance of natural products in gastric cancer therapy. The need for further research on the activity of single natural compounds isolated from the crude extracts in living organisms should be treated as an urgent future perspective.

## Figures and Tables

**Table 1:** The identity of the described plant species with their anticancer properties against the gastric cancer.

Plant Species	Active Compound	Cell Line	Bioactive Effect	Ref.
*Cordyceps cicadae*	Cordicepine	SGC-7901 MGC-803 HGC-27	1. Antiproliferative effects cell cycle arrest at S phase targeting PI3K/Akt pathway 2. Proapoptotic action ↑ cellular stress ↑ caspase-3 and PARP cleavage ↓ survivin expression 3. Antimetastatic effects ↑ epithelial markers expression ↓ mesenchymal markers expression	[18]
*Allium sativum*	Allicin	SGC-7901 MGC-803 BGC-823	1. Antiproliferative effects cell cycle growth at G2/M phase ↓ telomerase activity 2. Proapoptotic effects ↑ caspase-3 cleavage ↑ Bax/Bcl-2 ratio targeting p38/MAPK pathway	[19,20,21]
*Camellia sinensis*	Epigallocatechin gallate	SGC-7901	1. Antiproliferative effects ↓ EGFR and IGF-1R targeting Wnt/β-catenin pathway ↓ HDAC and DNMT expression 2. Proapoptotic effects ↓ ROS generation ↑ caspase-3 and PARP cleavage 3. Antimetastatic effects ↓ VEGF expression 4. Antiangiogenic effects ↑ ROS generation ↓ VEGF expression	[22,23,24,25,26,27,28,29]
*Cardiospermum halicacabum* (water extract)	Synthesized gold nanoparticles (CH-AuNP)	AGS SNU-5 SNU-16	1. Proapoptotic effects ↑ ROS generation ↑ Bax and caspase-3 expression ↓ Bcl-xl/Bcl-2 expression	[30]
*Plumbago zeylanica*	Plumbagin	AGS SGC-7901 MKN-28	1. Proapoptotic effects ↓ Akt and STAT targeting NF-κB pathway ↓ IAP1/XIAP expression ↓ Bcl2/Bcl-xl expression 2. Autophagic cell death induction 3. Antimetastatic effects ↓ NF-κB pathway ↓ VEGF expression	[31,32,33]
*Chrysosplenium nudicaule* (ethanol extract)	TTF and DTFG	SGC-7901	↑ apoptosis ↓ cell cycle	[34]
*Saussurea lappa*	Costunolide	SGC-7901 MGC-803 BGC-823	1. Antiproliferative effects cell cycle arrest at G2/M phase ↑ p53 and p21 expression ↓ cyclin B1 2. Proapoptotic effects ↑ caspase -3 and PARP cleavage ↑ Bax and Bak expression ↓ Bcl-2 expression 3. Antimetastatic effects ↓ VEGF expression	[35,36,37,38,39]
*Nigella sativa*	Thymoquinone	BGC-823 SGC-7901 MGC-803 HGC-27	1. Antimetastatic effects ↓ PI3K/Akt pathway ↑ epithelial markers expression ↓ mesenchymal markers expression ↓ JAK/STAT pathway ↓ VEGF expression 2. Proapoptotic effects ↓ JAK/STAT pathway ↓ cyclin D1, Bcl-2, survivin 3. Sensitizing cancer cells to chemotherapeutics	[40,41,42,43,44,45]
*Euphorbia lunulata*	Diterpenoids	SGC-7901/ADR	1. Antiproliferative effects cell cycle arrest at G2/M phase 2. Proapoptotic effects ↑ caspase-3 cleavage ↑ Bax expression ↓ Bcl-2 expression	[46]
*Euphorbia esula* (water extract)	Total extract	SGC-7901	1. Proapoptotic effects caspases expression ↑ Bax expression ↓ Bcl-2 expression	[47]
*Dioscorea bulbifera*(ethanol extract)	Diosbulbine B	SGC-7901	↓ cell proliferation	[48]
*Coptis chinesis*	Berberine	BGC-823 AGS SGC-7901 SNU-1	1. Antiproliferative effects ↓ c-myc cell cycle arrest at G0/G1 phase ↓ cyclin D1 and HNF4α 2. Autophagic cell death ↓ ERK/JNK/p38 MAPK pathway ↓ PI3K/Akt pathway 3. Proapoptotic effects PARP and caspase-3 cleavage ↑ caspases expression ↓ Bax/Bcl-2 ratio targeting p38/JNK MAPK pathway targeting NF-κB pathway 4. Antimetastatic effects ↓ MMP-3	[49,50,51,52,53,54]
*Stephania tetrandra*	Tetrandrine	HGC-27 BGC-823	1. Proapoptotic effects ↑ Bax/Bcl-2 ratio ↑ caspase-3 cleavage ↑ Apaf-1 2. Autophagic cell death ↑ LC3II and Beclin-1 expression ↓ PI3K/Akt/mTOR pathway 3. Sensitizing cancer cells to chemotherapeutics	[33,55,56,57]
*Piper longum*	Piperlongumine	SGC-7901 BGC-823 AGS MKN-45 MGC-803	1. Antiproliferative effects ↓ TrxR1 ↑ GADD45α ↓ cyclin B1 and Cdc2 expression cell cycle arrest at G2/M phase ↓ JAK/STAT pathway 2. Proapoptotic effects ↑ ROS generation ↑ p38/JNK pathway ↑ caspase-3 and PARP cleavage ↑ BIM expression 3. Antimetastatic effects ↓ JAK/STAT pathway ↓ MMP-9 and Twist	[58,59,60,61,62,63]
*Sophora* spp.	Matrine	BGC-823 SGC-7901 MKN-28	1. Antiproliferative effects cell cycle arrest at G1 and G2/M phases ↓ PI3K/Akt pathway ↓ uPA ↓ miR-93-5p expression ↑ AHNAK expression 2. Proapoptotic effects ↑ Fas/FasL expression 3. Autophagic cell death	[64,65,66,67]

**Table 2:** The chemical structures of the single active components of the extracts characterized by the inhibitory activity against stomach cancer, described in the review.

Plant	Bioactive Compound	Molecular Formula	Structure
*Allium sativum*	Allicin	C_6_H_10_OS_2_	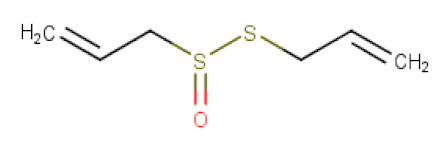
*Coptis chinesis*	Berberine	C_20_H_18_NO_4_^+^	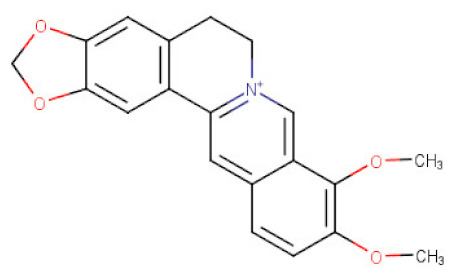
	Epiberberine	C_20_H_18_NO_4_	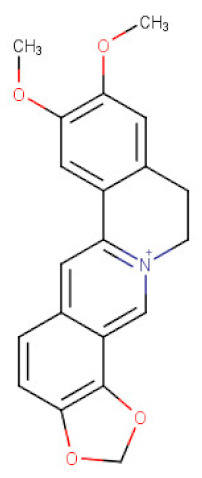
*Cordyceps cicadae*	Cordycepin	C_10_H_13_N_5_O_3_	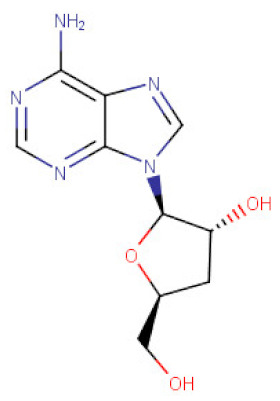
*Dioscorea bulbifera*	Diosbulbin B	C_19_H_20_O_6_	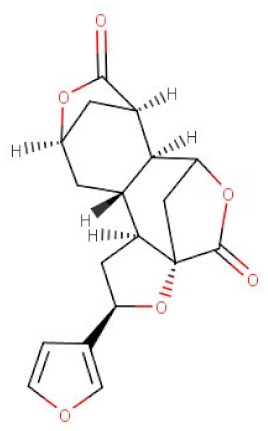
*Nigella sativa*	Thymoquinone	C_10_H_12_O_2_	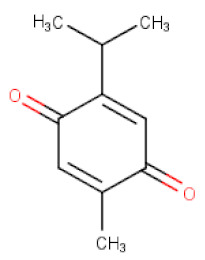
*Piper longum*	Piperlongumine	C_17_H_19_NO_5_	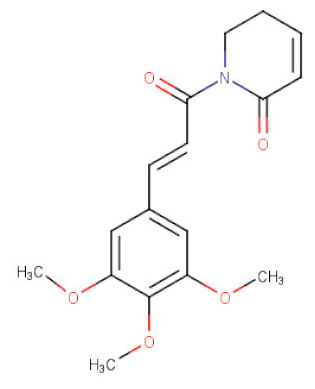
*Plumbago zeylanica*	Plumbagin	C_11_H_8_O_3_	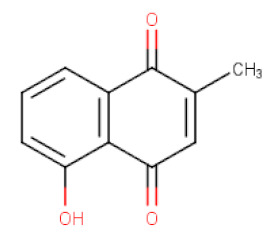
*Saussurea lappa*	Costunolide	C_15_H_20_O_2_	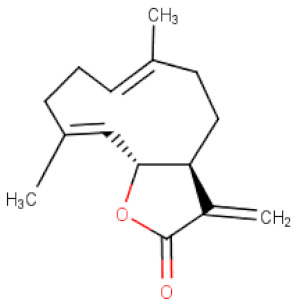
*Sophora* spp.	Matrine	C_15_H_24_N_2_O	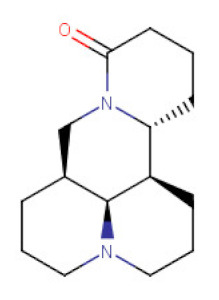
	Oxymatrine	C_15_H_24_N_2_O_2_	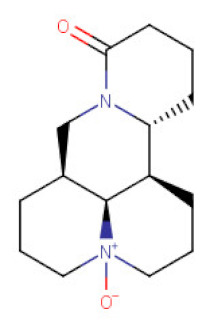
*Stephania tetrandra*	Tetrandrine	C_38_H_42_N_2_O_6_	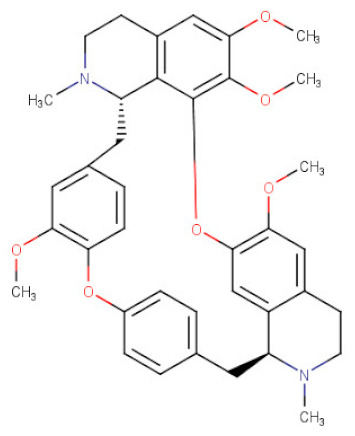

**Table 3:** The examples of in vivo studies on the activity of natural products against gastric cancer in xenograft mice.

Natural Product	Plant of Origin	Model	Mechanism of Action	Molecular Mechanism	References
Baicalein	*Scutellaria* spp.	Nude mice	Reduced tumor volume	Induced apoptosis, S phase arrest	[136]
Chaetocin	*Chaetomium* spp. fungi	Nude mice	Inhibited proliferation, induced cell cycle arrest (G2/M) and apoptosis	Inactivation of PI3K/AKT pathway	[137]
Cucurbitacin C	*Cucurbita* spp.	Female and male nude mice	Reduced tumor volume	Inhibition of STAT3	[138]
3,3′-diindolylmethane	Cruciferous plants	female nude mice	Inhibited tumor growth	Increase of LC3 levels (microtubule-associated protein light chain)	[139]
Dioscin	*Dioscorea* spp.	Nude mice	Induced apoptosis, cell arrest (S phase), inhibited tumor growth	Induced ROS generation and Ca^2+^ release	[140]
Formononetin-coumarin hybrid		Nude mice	Inhibited tumor growth	Inhibition of SIRT-1, Wnt/β-Catenin and AKT/mTOR pathways	[141]
Nitidine chloride	*Zanthoxylum nitidum*	Male BALB/cA nude mice	Reduced tumor volume	Decreased STAT3 and VEGF	[142]
Perillaldehyde	*Perilla frutescens*	Female BALB/c nude mice	Inhibited tumor growth	Increased caspase-3, p53, catepsin and LC3-II	[143]
S-allylmercaptocysteine	Garlic	Female BALB/e nude mice	Inhibited tumor growth	-	[144]
6-shogaol	*Zingiber officinale*	Athymic nude mice	Inhibited tumor growth		[145]

**Table 4:** The examples of clinical studies on the activity of natural products against gastric cancer in humans.

	Natural Product/Extract	Study Group	Dosage	Comments	Reference
Gastric cancer	Garlic extract and oil	3365 residents with high risk of gastric cancer	200 mg aged garlic extract and 1 mg steam distilled garlic oil for 7 years	Garlic supplementation was associated with a significantly reduced risk of incidence of gastric cancer and death	[146]
	*Rhus verniciflua* extract rich in flavonoids	An 82-year old woman	900 mg of extract for 5 months	Marked decrease in the polyploidy mass of the tumor and decreased lesion of the prepyloric antrum	[147]
	*Marsdenia tenacissima* extract	1329 patients (51–68 years old)	Injection: 40–80 mL/dose, 7–21 doses/session orally: 6–7.2 g/dose, 30 doses/session	Adjuvant therapy with anticancer treatment (typically folinic acid + fluorouracil + oxaliplatin) improved the response to chemotherapy and reduced thrombocytopenia, anemia, nausea, peripheral neurotoxicity and hepatic injury	[148]
	Curcumin from *Curcuma longa*	25 patients	500 mg/ day, 3 months	Histologic improvement of lesions	[149]
	*Aloe arborescens* rich in antraquinonic and anthracenic molecules	240 patients (58–79 years old)	10 mL/ thrice daily orally (300 g of leaves in 500 g of honey in 40 mL of 40% alcohol) 6 days before and during chemotherapy	Adjuvant therapy of metastatic solid tumor with 5-florouracil. Increased tumor regression	[150]

## Abbreviations

GC	Gastric cancer
WHO	World Health Organization
DNA	deoxyribonucleic acid
VEGF	vascular endothelial growth factor
IL-8	interleukin-8
bFGF	basic fibroblast growth factor
PD	ECGF- platelet-derived endothelial cell growth factor
FGFs	fibroblast growth factors
EGF	epidermal growth factor
EGF	CFC -epidermal growth factor-CFC
TGF-α	transforming growth factor alpha
AR	amphiregulin
TGF-β	transforming growth factor β
IGF II	insulin-like factor II
EMT	The epithelial–mesenchymal transition
NRP-1	neutrophilin-1
IL-1 α	interleukin 1-α
IL-6	interleukin 6
NF-κB	nuclear factor kappa-light-chain-enhancer of activated B cells
IC_50_	half-maximal inhibitory concentration
ECM	extracellular matrix
DAS	diallyl sulfide
DADS	diallyl disulfide
DATS	diallyltrisulfide
MAPK	mitogen-activated protein kinase
SH	sulfhydryl group
HSPs	heat shock proteins
CRP	C Reactive Protein
TLR	Toll-like receptor
EGCG	epigallocatechin-3-gallate
ROS	reactive oxygen species
AMPK	activated protein kinase
HIF-1α	Hypoxia-inducible factor 1
Bcl-2	antiapoptotic protein B-cell lymphoma-2
CytC	cytochrome C
PARP	Poly (ADP-ribose) polymerase 1
IGF-1R	insulin-like growth factor receptor
ERK1/2	extracellular signal-regulated kinase
Akt	RAC-alpha serine/threonine-protein kinase
STAT3	Signal Transducer and Activator of Transcription 3
AP-1	Activating Protein-1
DNMT	DNA methyltransferase
HDAC	histone deacetylases
CH-AuNP	gold nanoparticles
IKKβ	IKB kinase protein
IAP1	inhibitor of apoptosis 1
XIAP	X-linked inhibitor of apoptosis
TTF	6,7,3′-trimethoxy-3,5,4′-trihydroxy-flavone
DTFG	5,4′-dihydroxy-3,6,3′-trimethoxy-flavone 7-O-β-D-glucoside
PARP	poly ADP ribose polymerase
TQ	thymoquinone
5-FU	5-fluorouracil
CDDP	cisplatin
MDR	cancer multidrug resistance
PTEN	phosphatase and tensin homolog
BBR	berberine
LC3-II	Microtubule-associated protein
mTOR	The mammalian target of rapamycin
MAPK	mitogen-activated protein kinases
MMP	matrix metalloproteinases
PL	piperlongumine
TrxR	Three thioredoxin reductase
MA	matrine
OMA	oxymatrine
IL-21R	Interleukin-21 receptor
